# Genito-Urinary and Syphilitic Diseases

**Published:** 1900-01

**Authors:** Byron H. Daggett

**Affiliations:** Buffalo, N. Y.


### GENITO-URINARY AND SYPHILITIC DISEASES.

Conducted by BYRON H. DAGGETT, M. D., Buffalo, N. Y.

### RENAL CASTS—THEIR SIGNIFICANCE AND DETECTION.

LINSLEY, J. H., (*New York Med. Record*, October 21, 1899,) says there is no disease so frequently undetermined in its incipiency as chronic Bright's. It occurs about middle age in the apparently strong and robust. It is the most insidious of maladies and is often well advanced before detection or even before advice is sought.

The first symptoms are frequent nocturnal micturition (two or three times), slight dyspnea and mild digestive disturbances. Neuralgia will affect persons of a nervous temperament. The urine may be normal in color, specific gravity and reaction. Generally there is no albumin, occasionally a trace (1.40 of 1 per cent. by weight). The usual cloud of nearly transparent mucus may form after an hour or two. Place some of the deposit formed by the centrifuge under a microscope and hyaline casts, crystals of uric acid and oxalate of calcium, a few red blood cells, a few leucocytes and squamous and possibly round epithelia will be found.

The hyaline cells and blood cells are of the greatest importance. The casts are found in the renal tubules. The renal tubule begins in the Malpighian body. The renal tubule is 1.120 of an inch in diameter and consists of a loop of blood capillaries from the afferent vessel and is undeveloped in a delicate membrane and a single layer of flat epithelial cells. Within this envelope are found the convoluted, spiral and irregular tubules; larger arched and straight tubes, Henle's loop and excretory duct. The spiral, convoluted and irregular tubes are known to possess secretive power They are lined with the "rodded cell of Heidenhain." The cells of all the active tubules are flat and columnar.

The lining membrane of the Malpighian body or Bowman's capsule, normally, is a filter for water, and is an effective barrier to the transmission of all organic constituents. These are eliminated in the convoluted, spiral and irregular tubules by a process of true secretion. In health none of these will allow the passing of the albuminous constituents of the blood plasma.

If, however, there be impaired nutrition of the tubular walls, excessive pressure from the renal artery, or any degeneration of the basement membrane or lining cells, the blood plasma may pass through the walls of the tubules and coagulate in their lumina. This serous exudate passing into the uriniferous tubules forms a plug, which washed out by the urine forms the hyaline cast. The hyaline cast is the basis of several other casts, such as the epithelial, granular and blood casts. The cast may be loaded with leucocytes, bacteria or fatty lobules. Varieties are: hyaline, granular, epithelial, mixed, blood, fibrin, pus, bacterial, waxy and cylindroid. These may be narrow or broad. Broad casts have the greatest significance; hyaline casts indicate an advanced chronic interstitial nephritis.

Granular casts are the result of disintegration, and indicate a nephritis of some little standing with involvement of the parenchyma of the kidney.

Epithelial casts indicate acute parenchymatous inflammation.

Mixed casts have the same diagnostic value as the epithelial.

Blood casts indicate renal hemorrhage, due to traumatism or some destructive disease.

Pus casts are rare.

Fatty casts indicate an active degeneration of protoplasm.

Bacterial casts are found in septic nephritis and pyelonephritis.

Cylindroids, if from the kidneys, have the same significance as hyaline casts. If from the urinary tract, have no significance of renal disease.

Detection of casts: The sediment is put on the stage of a microscope, without a cover, and the examination made through a three-quarter or half inch objective and illumination of the specimen carefully made. After carefully examining the specimen under a low power, put on a cover glass and use a No. 5 or 7 objective. This will bring out the structure of the casts, if present, and epithelial cells and detect pus and blood cells as well as bacteria. The examination should always begin with the lower power.

